# From doubt to trust: Swedish mothers’ and counsellors’ experience testing a parenting programme for mothers exposed to intimate partner violence whose children have developed behavioural problems

**DOI:** 10.1177/1359104520931569

**Published:** 2020-07-10

**Authors:** Helena Draxler, Renée McDonald, Fredrik Hjärthag, Kjerstin Almqvist

**Affiliations:** 1 Department of Social and Psychological Studies, Karlstad University, Sweden; 2 Southern Methodist University, USA

**Keywords:** Parenting programme, intimate partner violence, feasibility study, behavioral problem, qualitative study

## Abstract

Many countries seek evidence-based interventions for children who have been exposed to domestic violence, and they frequently turn to interventions developed in the US and disseminated to Europe as a solution. Societal and cultural differences may, however, pose barriers to successful implementation. Project Support (PS), piloted in this study through social services agencies in Sweden, has shown positive effects in the US for families with children who have been exposed to intimate partner violence (IPV). The aim of this study was to investigate counselors’ and caregivers’ experiences when giving/receiving PS in Sweden.

The study was based on interviews conducted with 11 mothers and 13 counselors with experience in the programme. A thematic analysis showed three themes (Initial doubts, Confidence from positive change, and Flexibility- challenge for the organization) and the study adds information about obstacles for implementation of PS in Sweden. Cultural and organizational differences between Sweden and the US in practice and child-rearing attitudes are highlighted, as well as the importance of making adjustments while maintaining treatment fidelity, and promoting the dissemination of the approach.

The detrimental impact of parental intimate partner violence (IPV) on children’s psychological adjustment has been well established in numerous studies, with child-externalizing problems being a particularly prominent outcome (Vu et al., 2016). Reduced parental capacity, even after IPV has stopped, seems to be part of the explanation for children’s further negative development (Lieberman & Van Horn, 2005). Hence, it is necessary to find interventions and programmes that not only improve parents’ parenting capacity but also target children’s problems. For a successful outcome, the intervention needs to meet the mother’s individual needs and her whole situation (Levendosky et al., 2000). Several parenting programmes for families with children who have externalizing problems have been developed and parenting programmes such as Triple P and The Incredible Years have shown positive effects (Dretzke et al., 2009; Piquero, 2016). Components that are associated with positive outcomes include home visitations, enhancement of positive parent–child interactions, strengthening of parents’ emotional communication skills, and increase of parents’ knowledge of how to use time-out and how to act consistently (Kaminski et al., 2008; Lundahl et al., 2006).

Sweden has highlighted the need to support families exposed to IPV, and in 2007 it enacted a law requiring social services agencies to provide interventions for women exposed to IPV and their children (SFS 2001:453). Results of a Swedish evaluation of existing support programmes showed a need for interventions with more substantial effects on children’s psychological symptoms (Broberg et al., 2011). Project Support (PS) was selected to be tested in Swedish social services based on the positive effects shown in randomized control trials and systematic evaluations (Australian Centre for Posttraumatic Mental Health & Parenting Research Centre, 2013; Jouriles et al., 2009; Jouriles et al., 2010; McDonald et al., 2011). This report presents data from an initial pilot of PS in Sweden, examining aspects of feasibility when offered through Swedish social services agencies.

## Project Support (PS)

PS embraces the family’s contextual situation in the aftermath of recent IPV and targets the exposed parent and children at ages 3 to 9 years old. Thus, it includes social/emotional support for parents, such things as housing, financial and legal assistance, and law enforcement. PS also focuses on enhancing parents’ parenting skills, increasing parental sensitivity and warmth, and promoting pro-social child behavior. Services are provided in the families’ homes once a week for about 90 min. Roughly half of each session focuses on social and emotional support; the other half focuses on teaching and practicing specific parenting skills in role-play—skills such as attending, praise, listening and comforting, reprimanding and redirecting, and using time-outs. The skills are cumulative and taught in progression, beginning with skills to increase the frequency of positive parent-child interactions and to shape and reward pro-social behavior, followed by skills for dealing with misbehavior. The parent practises each skill, and progresses to the next skill only after demonstrating mastery of the previous skill. Because families vary in their need for additional feedback and practise, the length of the programme can differ across families (Jouriles et al., 2001).

PS was designed to be appropriate for any parent or other family member who is a caregiver of the child; however, in past evaluations of the programme only mothers and their children participated. The mothers that received PS demonstrated reductions in harsh parenting, children’s externalizing problems decreased compared to families that received services-as-usual (Jouriles et al., 2001, 2009), and mothers were less likely to return to their violent partner (McDonald et al., 2006). The positive effects were sustained through a 20-month follow-up period. Positive effects such as reductions in harsh parenting and use of violence have also been shown for parents at a child welfare agency for child physical maltreatment. PS also seems to reduce children’s symptoms of psychopathy (Jouriles et al., 2010; McDonald et al., 2011). A small pilot study of PS in Sweden, partly with data from mothers from this study, indicates similar reductions in child symptoms and improved parenting in mothers, suggesting that the pattern of effects found in the US may generalize to Sweden (Draxler et al., 2019).

## Implementation considerations

The intent of a feasibility study is to answer questions such as: To what extent do the stakeholders find the intervention appropriate for their purpose? Will the intervention suit the organization? Are the outcomes sustained? Aspects of feasibility also require knowledge about acceptance and adaptation needs (Bowen et al., 2009).

Parenting programmes from the US have successfully been implemented in Europe (Axberg & Broberg, 2012). However, some implementers have found it necessary to make programme adjustments to facilitate successful uptake, primarily by removing or adjusting skills designed to correct misbehaviors (Hasson et al., 2014; Rahmqvist et al., 2014). Swedish experiences from implementing methods for parents exposed to IPV have also shown that organizational structure, processes for referring families to interventions, and counselors’ commitment play important roles in the implementation process and outcomes (Källström & Grip, 2019). In addition, barriers such as practical difficulties (transportation, childcare, location, etc), time constraints (work, many children, etc), lack of information, and local availability of services play an important role receiving parenting interventions (Koerting et al., 2013).

Differences in laws and regulations, organizational structures, and the cultural contexts of the US versus Sweden (World Value Survey, 2020) raise questions about the feasibility of implementing PS in Sweden. Hence, the aim of this study was to investigate the experiences of caregivers and counselors in a pilot of PS in Sweden.

## Method

### Procedure

Information about the initial pilot of PS and an invitation to take part in an assessment of its feasibility in Sweden were provided to a range of social services providers as part of a national evaluation of interventions for children exposed to parental IPV (Almqvist & Draxler, 2016; Broberg et al., 2015). PS was one of four interventions included in the national evaluation. Social services agencies in four mid-sized municipalities and a domestic-violence shelter in a large city volunteered to participate. The counselors in these organizations (with their supervisors’ support) provided written consent and took part in a training course in Sweden. They also received consultation calls and regular clinical supervision for their early PS cases with agency clients, as part of their training. In accordance with their ordinary routines, the participating services decided which families were appropriate to take part in PS, as long as the families met the inclusion criteria: having experienced IPV and having a child within the age range (3–9 years) who was exhibiting behavioral problems.

Families who received PS services also provided informed consent to the social services agency providing their services, regarding participation in the feasibility study. Although any caregivers of a child were welcome to participate, just as in the US, only mothers exposed to IPV (i.e., no fathers) chose to participate in PS and in the study. Because this was the first attempt to implement PS in another country, it was important to monitor service delivery and counselors’ and mothers’ perceptions of the programme, to minimize risk of low-fidelity implementation or counselor or mother dissatisfaction. Therefore, the first author conducted interviews with counselors and mothers who had participated in at least five PS sessions. Because of the geographic spread of the participating agencies, and practical and logistical challenges in scheduling the in-person interviews in the families’ homes, the range of sessions that the various participants had completed when the interviews were conducted was from five to fifteen. All interviews were conducted over an eight-month time span. The mothers had legislative, regulatory, and insurance coverage from the respective participating organization. The study was approved by the Swedish Ethical Review Authority, nr. 2013/115 (and was made in accordance with the Declaration of Helsinki [WMA, 2013]).

### Participants

Families in the study had been referred to the social services agencies by schools, the police, other social services agencies (e.g., domestic-violence shelters), or had sought social services on their own initiative. Some of them had previously received other interventions that they had not found to be sufficiently helpful for their children.

#### Counselors

Ten female and three male counselors (age range: 33–62 years, *M_age_* = 47 years) took part in the study. Most were experienced in their profession (range 5–40 years, *M* = 16 years) and 12 had a college degree in the social or behavioral sciences. Prior to the PS training, participating counselors stated they were accustomed to providing non-manualized support to parents in need of social services, following from their college training (Broberg et al., 2015). They generally followed the clients’ lead regarding family problems, providing emotional support to alleviate distress, and providing education and guidance on parenting.

All counselors took part in the PS training during a year which consisted of a three-day workshop, followed by a booster workshop, and regular supervision conducted by the PS originators (McDonald and Jouriles) given in English. When interviewed, two of the counselors had provided services to two families; the remainder to one family each.

#### Mothers

Eleven mothers were included. Records of their age were not collected; however, they were estimated by the counselors to be between 20 and 45 years old. Six mothers were employed while five relied upon various forms of public financial assistance. There were between one and five children in each family. The mean age of the target child in the family (the child identified as having behavioral problems and falling within the 3 to 9 age eligibility criteria) was 6 years (six males, five females). Three mothers were living in a domestic shelter with their children when they took part in the study. One child alternated living with both parents, who were separated; the remainder lived with their mothers. The mother had sole custody of five of the target children; the remainder had joint custody with the child’s biological father. Three mothers informed about the study declined to participate due to an upcoming move of residence or because they preferred another service (e.g., child-only services).

#### Treatment fidelity

For each session, the counselors completed a structured fidelity form assessing adherence to the method according to manual and training, satisfaction with their own efforts, and parental response to the intervention. Responses to the items were made on a 10-point scale, with 10 indicating the highest level of adherence or satisfaction and one indicating the lowest level. In the 113 sessions that were conducted, counselors rated their own fidelity to the intervention to a mean of 7.15 (*SD* = 2.25) and their satisfaction with their own efforts to a mean of 7.71 (*SD* = 1.68). counselors rated parents’ adherence to a mean of 7.99 (*SD* = 1.80). In addition, the counselors recorded which parenting skill had been the focus in each session, and indicated the extent whether the skill had been introduced, practised, and/or reviewed. All counselors reported practise with the skills focusing on responsive parenting and promotion of pro-social behavior, while only one counselor reported practise of the skills to respond to child misbehavior.

### Interviews and analytic approach

All 24 interviews were made using semi-structured interview guides (Appendix 1 and 2). Interview questions were specifically focused on aspects of feasibility such as: Acceptance, Satisfaction, Training, Conditions for Implementation, Practical Realization of the Programme, and Adaptation Needs. Four of the caregiver interviews were conducted in the mothers’ homes and seven at the participating social services agency office. The interviews with the counselors were conducted at their workplaces, except for one interview that was done in the counselor’s home, at the counselor’s request. The interviews were 35 to 75 min long, audio-recorded, and transcribed verbatim for coding. Thematic analysis of the data was based on methods of Hayes (2000) and Braun and Clarke (2006).

#### Thematic analysis of interviews

The first author read the transcripts of the mother interviews several times and listed meaningful units as initial codes. Codes that seemed to cover the same general area of feasibility aspects were combined. This process yielded fourteen potential themes. The interview transcripts were then read through again to identify information such as statements or specific wordings, to validate each potential theme, as a way of being meticulous to the transcripts. During this phase, the transcripts were read through several times, and preliminary conceptualisations of the themes were discussed with the third and last author. The last author also read randomly selected interviews and independently coded them for comparison against the first author’s analysis, to assess for discrepancies. The same process was used to code the counselor interviews, and this process yielded nine potential themes.

Further, decisions on how to establish the final themes based on all the potential themes were continuously discussed among the authors, and several revisions and clarifications were made. Three final themes emerged from the analysis of both the mothers’ and counselors’ experiences, and illustrative quotes were taken from the empirical material (see Braun & Clarke, 2006, and Hayes, 2000 for additional background) (see Table 1).

**Table 1.** First- and second level synthesis of thematic content of mother and counselor interviews.

Potential themes	Final level of themes
M1 Personal barriers	M1, M2, M3, C1, C2, C3, C4 Initial doubts
M2 Role-play	
M3 Adaptations and adjustments	
M4 Thoughts about the future	
M5 PS suits all parents	
M6 Early response/ consequences	
M7 Experience from previous interventions	
M8 Changes in the child’s behavior	
M9 Mothers’ changes and views of their parental role	M5, M6, M7, M8, M9, M10, C5 Confidence from positive change
M10 Changes in mother–child interaction	
M11 Intensity and regularity	
M12 Clarity and structure with space for customization	
M13 Adaptations and adjustments	
M14 Counselors role and contacts with them	
C1 Implementation of PS	
C2 Personal limitations	M11, M12, M13, C6, C7, C8, C9 Flexibility- challenge for the organization
C3 Mothers’ limitations	
C4 Adaptation needs/criticism	
C5 Strengths of PS	
C6 Difficulties in recruiting cases	
C7 The organization’s resources	
C8 Organizational form	
C9 Legislation	

“M” denotes mother interview data; “C” denotes counselor interview data.

## Results

The following three themes emerged from the analysis: Initial doubts, Confidence from positive change, and Flexibility - challenge for the organization.

### Initial doubts

Counselors as well as mothers expressed that they initially found it hard to work with the programme. Counselors reported difficulties in explaining and providing a rationale for aspects of PS. They met resistance from mothers and found it hard to motivate them and to individualize and tailor the training. One mother was disappointed that she should start with praising her child instead of focusing on misbehavior and said: “But I need help with keys to managing X’s anger as well, because I don’t know how.”

Mothers and counselors also described that before the training they felt insecure about the way the skills were taught and how the children would respond. Counselors expressed a lack of familiarity with skills-training approaches involving behavioral practise. About the practise activities, one mother said: “I think I was scared somehow. That I wouldn’t be able to . . . and if I wasn’t able to, it would stir up so many emotions—I don’t know my own child.”

Counselors tended to fall back on their general way of working with mothers who have been exposed to IPV and focused on providing support and on external factors, such as demands to manage a new life situation. One counselor reported: “We can see that we make mistakes when we fall into the usual social work . . . fall into this trap and focus too much on the problems.”

During the initial training workshop and their first experiences with PS, counselors expressed strong discomfort with the potential use of the Time-out and Ignoring skills. In the interviews with the counselors who had just begun implementation, most counselors still expressed a strong dislike. They considered it unethical to give children a consequence such as a time-out for unwanted behavior and regarded ignoring as a way of ostracizing the child. The counselors described those restricting parenting skills as against their own cultural values or ideology and in conflict with the UN Convention on the Rights of the Child and with Swedish laws which prohibit both corporal punishment and psychological violation of children. Moreover, the counselors expressed that they were afraid the mothers would exceed limits and misuse the restricting parenting skills:

It’s just going to be a penalty and they [the children] will feel even worse . . . and it’s going to be like this for the rest of their lives. I think things like this will be stigmatizing . . .

### Confidence from positive change

When counselors and mothers gained experience in role-plays and more understanding of PS, their trust increased in the method and in their own capacity for success. They considered the parenting skills to have a great impact, and indicated that children’s change came surprisingly quickly. They described that behavioral problems decreased while pro-social behavior improved. Participants also described changes in the mothers, such as improved confidence, decreased harsh parenting, and a better understanding of children’s reactions. As the mothers became more responsive and warmly engaged with their children, their mental health improved and the mothers said they intended to continue using the PS parenting skills so it would become a natural part of their interaction with their children.

A mother said:

I believe that all parents should do this even if they haven’t been exposed to violence, because I think children, in turn, become better—and it might not be so much fighting in schools and all, if all parents do this attending and praise.

A counselor expressed it in this way:

When mothers start to see their children, and receiving something- that’s joy. It isn’t just a burden, it isn’t just a bad conscience, it isn’t just ‘I’m a bad mother. ‘ But they know somewhere that I’m okay as I am, and my kid loves me. `

Both counselors and mothers expressed that focusing on the parent’s potential for agency in helping their children is one of the most important components of PS. They saw PS as being different than other interventions, and the caregivers got more confidence to actually influence their own family situation. A mother expressed hopefulness:

When we tried this (PS), it’s something she and I did ourselves as well. There’s a big difference . . . and I can do it as many times as I want. It was difficult to get anything as concrete as that from the mental health system.

The counselors appreciated the two parts of PS - social and emotional support and parenting skills training, and the counselors often acted as a supplement to the mothers’ poor social support systems. The role-plays gave them the opportunity to work with actual behaviors. One counselor stated: “There’s a big difference, and we haven’t had the opportunity and the access to all parts before.”

### Flexibility-challenge for the organization

Counselors described difficulties in establishing PS within their organizations - difficulty in getting referrals, the resource demands of the programme, and a lack of support from others (manager, colleagues, collaborators etc.) in the organization. Mothers and counselors wished to work intensively on a regular basis, and most of the mothers were self-supporting and received no compensation for taking part in PS during daytime. All participants also saw the benefit of delivering or receiving the programme in the families’ homes at times that suited the family. However, this required restructuring the organization and one counselor said: “Then I’d have to work at seven in the evenings or on weekends, and it’s not possible for me and my family. I’m not working those hours.”

The counselors stressed that PS is an intervention for the caregiver and not for the child, despite its focus on reductions in child externalizing problems as an outcome. The counselors explained their view that this means that one caregiver with child can take part in PS without the other custodian’s consent and approval. As many parents in Sweden have joint custody and both custodians’ consent is needed for children to take part in treatment, they saw this distinction as important.

## Discussion

The aim of this study was to examine Swedish counselors’ and parents’ experiences and opinions about the PS programme for caregivers who have experienced IPV. The results indicated that both counselors and mothers in general appreciated the method and the support it had given to change parenting behavior and to decrease children’s behavioral problems. However, both caregivers and counselors initially expressed resistance concerning the use of role-plays for practicing the skills, but they also described how their attitude changed after overcoming their doubts and trying the intervention. Counselors highlighted the need for cultural adaptation concerning child rearing, especially regarding parenting skills for responding to child misbehavior, and described organizational challenges within social services agencies. The themes that emerged are consistent with those of others who have conducted research on implementation of new methods from the US for mothers exposed to IPV within Swedish social services (Källström & Grip, 2019).

In this study, mothers and counselors appreciated the fixed structure of PS and that individual adaptation and flexibility were possible. The need to adapt PS to each family also constituted a challenge for the counselors who needed a high competency concerning child development and thorough knowledge about the family. This, coupled with the fact that this was the first time they were delivering PS to clients, may perhaps explain some of their initial doubts. The results are well in line with those of Koerting et al. (2013) where mothers and counselors expressed similar concerns about their own personal capacity. Koerting et al. (2013) also described other important aspects similar to our findings: for example, organizational and legislative limitations, organizations’ prospects for working with an intervention even if it causes inconvenience, the importance for mothers to establish emotional closeness with the counselors, and individualization for facilitating implementation. The counselors’ competence is crucial, as are regular support and supervision of their work (Sewell, 2018).

### Implementation adaptations: Implications

For acceptance and sustainability of transferred programmes, adjustments seem to be important (Hasson et al., 2014). When it comes to PS, this study highlights that systematic methods for recruiting or referring families for PS services need to be developed in Sweden. As well, resources within the social welfare organizations need to be allocated and prioritized so that the services are adequately supported. This aligns with previous implementation research highlighting the need for organizational support and a structure for counselors’ work schedules (Koerting et al., 2013; Källström & Grip, 2019).

Parenting skills that include positive reinforcement are generally more accepted than those that include punishment or extinguishing methods (Axberg & Broberg, 2012; Kling et al., 2006; Rahmqvist et al., 2014). The modifications of the restricting skills that were made in other programmes during the Swedish implementation process were made despite the fact that parents who adopt restricting parenting skills generally decrease their harsh parenting style and find alternatives to hitting their children (Tully, & Hunt, 2016). Counselors in this study expressed concerns whether the restricting skills in PS are in conflict with Swedish law and whether they could be considered to as psychological violation of children, which has been banned in Sweden for many years. The counselors’ fidelity check registrations showed that they did not practise the final skills that were designed to teach parents how to respond to misbehavior. This might be because the restricting parenting skills are deviant from counselors’ sociocultural norms (World Value Survey, 2020), but organizational barriers could also cause the practise. The restricting skills are at the end of the programme and implementation of a new treatment requires, organizations that prioritise the method and makes it possible for counselors to have a long contact on regular basis (Källström & Grip, 2019; Koerting et al., 2013).

### Lessons learned for further implementation

Before further implementation of PS in Sweden, it may be useful to work with the programme originators to make cultural adjustments concerning the parenting skills, and also to clarify organizational conditions that are necessary for delivering PS. According to the originator of the programme, resistance among counselors to the restricting parenting skills because they are perceived as too harsh was not known from dissemination of PS in the US (R. McDonald, personal communication, March 26, 2020). Sweden deviates in several aspects from other countries regarding philosophical, political, and religious ideas and when modifying the intervention, it is important to not change the effective components (Fixsen et al., 2005; Wold Values Survey, 2020).

The conceptualisation of, for example, Time-out or other skills for responding to misbehavior wary depending on psychological theory (Dadds & Tully, 2019) and it is important to use descriptions appropriate and acceptable to the Swedish context.

The results from this study indicate that a script or list of frequently asked questions that counselors could use might increase their confidence and efficacy. Doubts about practicing through role-play also need to be addressed. It might be helpful to rename the role-plays simply as “practise,” since the concept of role-play seems to elicit performance anxiety. In addition, previous findings stress that counselors could benefit from motivational skills in order to improve conditions for both compliance and effect (Koerting et al., 2013).

### Limitations

This is a qualitative study with a limited sample in both number and gender. Although generalisability of the results is therefore limited; the aim was to collect information that can help address concerns about further implementation and research.

The counselors were experienced and interested in new interventions in this field. The recipients of PS, who were all mothers, were help-seeking and put a lot of time and effort into their participating in the intervention and the study. Social desirability and selection biases may have contributed to the results. The interviews were made during treatment and it would be valuable to see how counselors and mothers experienced PS after they finished treatment or to see counselors’ views of PS after experiencing more work with more families. In addition, since children can experience events differently from adults (Lieberman & Van Horn, 2011), results may have differed if the children had been interviewed as well.

## Conclusion

The results indicate that PS was perceived by counselors as well as mothers as a helpful and effective programme, despite considerable initial doubts. The informants showed resistance to practise role-plays, an unusual technique in Sweden, which needs to be considered in future training for counselors. Both counselors’ negative perceptions of the restricting parenting skills, and that those skills were not taught to the parents, indicate the importance of cultural adaptation and organizational conditions. Mothers described becoming more confident in their parenting and a decrease in their children’s behavioral problems, and counselors described feeling more competent.

## Supplemental Material

Appendix_1_and_2 – Supplemental material for From doubt to trust: Swedish mothers’ and counsellors’ experience testing a parenting programme for mothers exposed to intimate partner violence whose children have developed behavioural problems Click here for additional data file. Supplemental material, Appendix_1_and_2 for From doubt to trust: Swedish mothers’ and counsellors’ experience testing a parenting programme for mothers exposed to intimate partner violence whose children have developed behavioural problems by Helena Draxler, Renée McDonald, Fredrik Hjärthag and Kjerstin Almqvist in Clinical Child Psychology and Psychiatry
