# Supplementary material for: From doubt to trust: Swedish mothers’ and counsellors’ experience testing a parenting programme for mothers exposed to intimate partner violence whose children have developed behavioural problems
Source: Clin Child Psychol Psychiatry. 2020 Jul 10;25(4):972–83. doi: 10.1177/1359104520931569 (PMC7528546; doi:10.1177/1359104520931569)
Supplement: Appendix_1_and_2 – Supplemental material for From doubt to trust: Swedish mothers’ and counsellors’ experience testing a parenting programme for mothers exposed to intimate partner violence whose children have developed behavioural problems [file Appendix_1_and_2.pdf]

Appendix 1

Interview guide: Parent

1. Acceptance

• How has it been for you and your child taking part in Project Support?
  Is there something that you find has been particularly good?
  Is there something that you find has been not so good?
  Do you feel that there is something missing?
  Is there something about taking part in PS that you feel discomfort about?

2. Satisfaction

• Did you and your child receive the help that you wanted?
  Were there things or situations you needed help with that PS could not fulfil?
  What has been the biggest help for you?
  What has been the biggest help for your child?
• Have you changed by taking part in PS?
  How?
• Have your child changed by taking part in PS?
  How?
• Have you, at any time, experienced "a turning point", or something that is of special importance to you or your child?

3. Conditions for Implementation

• Would you recommend PS to other parents and children?
• Are there situations when it is not appropriate to work with PS?
  Can you give an example?

4. Practical Realization of the Programme

• Have there been any difficulties for you to attend and engage in PS?
  What parts and why? (Invent all aspects; practical, travel, disruptions; child does not want to etc.)
• Do you think you will use things that you have learned through PS when you and your child no longer take part in the method?

5. Adaptation Needs

• Are there any particular parts/elements of PS that have not worked so well?
  Does the method need to be adjusted in some way to work better?
  Can you give suggestions how?

6. Final questions

• Have you received any other intervention for you and / or your child before?
  If yes, what advantages and disadvantages did you experience from that support compared to PS?
• Have you received any other support while you and your child were involved in PS?
  Has your child received any other kind of support?
• Is there anything you want to tell the researchers about how it was for you taking part in this this study and how it was done?

Appendix 2

Interview guide: counsellor

1. Acceptance

- Do you think Project Support (PS) meets the needs for your clients?
  - More specifically - violent parents and children with behavioural disorders?
- What do you think about working with PS?
  - Is there anything about the PS method that you feel hesitant about?
  - Do you feel satisfied about working with PS?
  - Do you think that the way of working with PS feels good?
  - Is there something concerning the PS method that you are doubtful about?

2. Training

- How have parents and children reacted to receiving PS?
  - Is there something that you think has been good concerning working with PS?
- How have parents and children reacted/responded when receiving PS?
  - Any specific/particular positive moments?
  - Difficult or unnecessary parts/elements?

3. Conditions for Implementation

- Do you think that PS is suitable for most parents and children in this situation?
  - Is PS better or less suitable for the corresponding target group?
- Are there situations where it would not be appropriate to work with PS?
  - Can you give an example?
- Have parents who did not want to work with PS justified why?
- Have there been any difficulties working with PS?
  - What difficulties? (Invent all aspects; practicalities, disruptions, lack of consensus, legal, cooperation with others, etc.)

4. Practical Realization of the Programme

- Will you continue to work with PS even when this project has ended?
  - Will more practitioners from your team learn the method?
  - Do you have the support from management level/manager to continue working with PS?
  - Do you see any obstacles to continuing to work with the method?

5. Adaptation Needs

- Are there any parts or elements of PS that you think do not work so well?
  - Does the method need to be adjusted in some way to work better?
  - Can you give some suggestions how?
